# Probing the elastic limit of DNA bending

**DOI:** 10.1093/nar/gku735

**Published:** 2014-08-13

**Authors:** Tung T. Le, Harold D. Kim

**Affiliations:** School of Physics, Georgia Institute of Technology, 837 State Street, Atlanta, GA 30332, USA

## Abstract

Sharp bending of double-stranded DNA (dsDNA) plays an essential role in genome structure and function. However, the elastic limit of dsDNA bending remains controversial. Here, we measured the opening rates of small dsDNA loops with contour lengths ranging between 40 and 200 bp using single-molecule Fluorescence Resonance Energy Transfer. The relationship of loop lifetime to loop size revealed a critical transition in bending stress. Above the critical loop size, the loop lifetime changed with loop size in a manner consistent with elastic bending stress, but below it, became less sensitive to loop size, indicative of softened dsDNA. The critical loop size increased from ∼60 bp to ∼100 bp with the addition of 5 mM magnesium. We show that our result is in quantitative agreement with the kinkable worm-like chain model, and furthermore, can reproduce previously reported looping probabilities of dsDNA over the range between 50 and 200 bp. Our findings shed new light on the energetics of sharply bent dsDNA.

## INTRODUCTION

Double-stranded DNA (dsDNA) can bend, twist, stretch and adopt various structures under thermal excitation (1–4). Despite this wide range of thermal fluctuations, mechanical properties of DNA at large length scales can be well described by the worm-like chain (WLC) model (5). According to this model, directional change in the chain contour costs energy quadratically dependent on bending angle. Chain stiffness can be described by the persistence length above which the chain directional correlation becomes negligible (6). The persistence length of dsDNA has been estimated to be 45–53 nm (132–156 bp) by various methods (7–14) and shown to be largely independent of monovalent salt concentration above 20 mM (15–17).

Strong bending of dsDNA, which refers to deflection of larger than ∼2.4° between adjacent base pairs (or equivalently, one turn per persistence length), occurs in transcriptional repression, nucleosome formation and viral DNA packaging (18). Since the WLC is valid only within the elastic limit of dsDNA, the actual bending energy of dsDNA in such processes may deviate from the WLC prediction. The free energy cost of dsDNA bending can be experimentally determined by measuring the efficiency with which a linear dsDNA can be ligated into a circle. By comparing the rates of circle and dimer formation in the ligation reaction, one can obtain an effective molar concentration of one end of the DNA around the other end, which is known as the J factor (11). Using this ligase-dependent cyclization assay, the J factors of dsDNAs shorter than 150 bp were measured to be several orders of magnitude higher than the WLC model predictions (19,20). In support of this result, an Atomic Force Microscopy (AFM) study also revealed more frequent sharp bends in the dsDNA contour than the WLC model (21). However, the result of the cyclization study could be an artifact due to high ligase concentrations used (22), and the result of the AFM study appears to be an artifact of data processing (23).

In a recent study, Vafabakhsh and Ha used single-molecule Fluorescence Resonance Energy Transfer (FRET) to measure the J factors of short dsDNAs (24). In this method, DNA loop formation can be detected without external agents such as protein or a bead that can bias the equilibrium looping probability of dsDNA (25–27). They found that J factors in the range between 65 and 110 bp determined from looping kinetics were a few orders of magnitude higher than the WLC model prediction. The results from this study suggest a significant departure of dsDNA from either the WLC model or 45–53-nm persistence length. However, other experimental factors could have led to an overestimation of the J factor: (i) using synthetic oligos may introduce mismatched base pairs (28), (ii) high salt conditions (1 M sodium or 10 mM magnesium) can increase DNA curvature and flexibility (29–32) and/or (iii) long sticky ends used in the experiment can increase the apparent looping probability (33,34). (i) can be addressed by using PCR-based DNA assembly (28,35,36), but (ii) and (iii) cannot be easily addressed because lowering salt concentration or shortening the sticky ends severely reduce the frequency of looping events observable by FRET for short DNA molecules (24,35).

**Figure 1.**
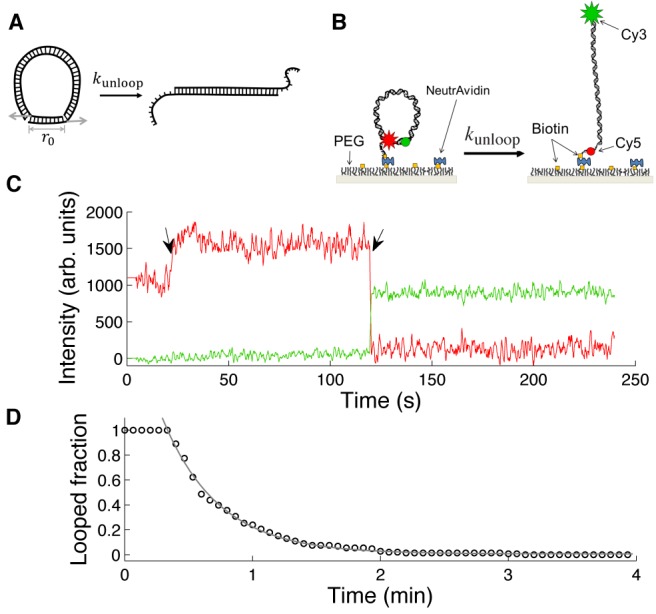
Loop breakage assay. (**A**) The shear force exerted on the linker duplex by the loop. The force exerted in the shear direction (gray arrows) accelerates dissociation of the linker duplex according to the Bell relationship (Equation (5)). (**B**) DNA design. A short DNA with sticky ends can be captured in the looped state when the two sticky ends are annealed. The looped state (left) and the unlooped state (right) correspond to high FRET and low FRET, respectively. For single-molecule experiments, DNA molecules are immobilized through a biotinylated end to a PEG-coated glass surface. In [Na^+^] = 2 M, a significant fraction of molecules exist in the looped state. Decreasing [NaCl] from 2 M to 50 mM by flow induces breakage of DNA loops. (**C**) A representative time trace of Cy3 (green) and Cy5 (red) intensities from a single molecule. The change in salt concentration causes an increase in the Cy5 intensity due to an unknown reason (marked by a black arrow). Upon loop breakage, Cy5 intensity drops, and Cy3 intensity jumps (marked by a black arrow). (**D**) The time decay of the number of dsDNA loops upon salt concentration drop. The molecules begin to unloop shortly after perfusion of 50 mM [Na^+^] buffer. The decay curve is fitted with a single exponential function to extract the lifetime of the DNA loop.

In this paper, we take a different FRET-based approach to test the WLC model at short length scales. The key idea is that stability of end-to-end annealed DNA loops is highly sensitive to loop size due to internal bending stress as depicted in Figure 1A. In our FRET assay, the looped state of a dsDNA is stabilized by formation of a transient linker duplex of ∼10 bp between its sticky ends. The lifetime of this linker duplex depends on the shear force exerted along its helical axis by the looped DNA. Since different DNA models make different predictions about how this shear force depends on the loop length, we can experimentally test these models by measuring linker lifetime versus loop size.

Our unlooping-based approach has unique capabilities that complement the ligation-based or FRET-based J factor measurements: (i) unlooping rates can be measured with good statistics in moderate salt conditions where looping of short dsDNA rarely occurs; (ii) only the molecules that were able to loop are followed in the loop breakage assay, which automatically filters out dysfunctional molecules and (iii) the unlooping rate is related to the shear force, which is easier to compute than the J factor.

Using this unlooping assay, we measured the lifetime of small DNA loops as a function of loop size in the strong bending regime. We found that the loop lifetime decreases with decreasing loop size, indicative of increasing bending stress. The bending stress, however, ceased to increase elastically below a critical loop size, reminiscent of a structural transition in dsDNA. The critical loop size increased in the presence of magnesium, indicating the role of divalent ions in softening dsDNA. We show that our data cannot be explained by a continuous polymer model with a single flexibility parameter, but instead calls for a model with an additional internal state such as a kinkable worm-like chain (KWLC) model. Using such a model, we can estimate the free energy of kink formation to be larger than $\sim18k_B T$, and $\sim12k_B T$ with 5 mM magnesium. We also show how the apparent discrepancy between previous J factor measurements can be quantitatively resolved by our KWLC model.

## MATERIALS AND METHODS

### Materials

The DNA molecules used in this unlooping assay have a double-stranded part with variable length from 37 bp to 189 bp and 13-nucleotide (nt) long single-stranded complementary overhangs (sticky ends). One overhang contains Cy3, and the other contains Cy5 and biotin (Figure 1B). The sequences of these overhangs are ATAG/iCy5/GAATTTACC, where /iCy5/ represents the internally labeled Cy5, and GGTAAATTCACTAT with the underlined ‘A’ inserted as a spacer opposite to iCy5 to increase the likelihood of base pairing around iCy5 that interrupts the backbone. All DNA molecules are derived from a master sequence that is ∼50% in GC content and does not have curvature-inducing patterns such as GGGCCC or A-tracts. The master sequence was constructed by annealing the ends of two 113-nt long single-stranded DNAs over a 16-nt region and extending their 3’-ends using DNA polymerase. The 210-bp master DNA was purified by gel electrophoresis, and PCR amplified with dangling-end primers to generate DNAs with common terminating sequences. The annealing location of one of the primers was varied to generate DNAs with different lengths. These PCR products were used as templates in another round of PCR to incorporate fluorescent labels and a biotin as previously described (35,36). Strands were exchanged between these PCR products to obtain the final DNA constructs for our experiment. Detailed sequences can be found in the Supplementary Information.

### Single-molecule unlooping assay

The DNA molecules were immobilized on a Polyethylene glycol (PEG)-coated glass surface through NeutrAvidin–biotin interaction. The immobilized molecules were excited by the evanescent wave of a 532-nm laser (NT66–968, B&W Tek, Newark, DE, USA) totally internally reflected through a high numerical aperture (NA) objective (UApo N 100×/1.49, Olympus). The power of the 532-nm laser was ∼5 μW when measured after the microscope objective before reaching the critical angle of incidence. For a split view of Cy3 and Cy5 images, the fluorescence image was split into the Cy3 and Cy5 channels outside the microscope and relayed onto an EMCCD (DU-897ECS0-# BV, Andor). A lab-written C program was used to view and save live images from the CCD. The raw image data were processed by MATLAB to generate single-molecule time traces of Cy3 and Cy5 intensities. In the loop breakage assay, immobilized DNA molecules were first incubated in 2 M NaCl buffer for up to an hour to generate looped molecules. We then introduced the imaging buffer (5 mM PCA, 100 mM PCD, 1 mM Trolox) that contains 2 M NaCl to start image acquisition. After 20 s, new imaging buffer with 50–200 mM NaCl was perfused into the imaging channel at a flow rate of 75 μl/min, which corresponds to ∼1 cm/s in flow velocity through the channel. The typical dimension of the channel cross-section is 0.075 mm × 2.0 mm. We recorded the times it takes for molecules to unloop from single-molecule time traces, built the survival time histogram and fitted it with a single exponential function to extract the linker lifetime.

### Force calculation for different DNA models

To calculate the shear force, we used umbrella sampling to generate the radial probability distribution $P\left( r \right)$. dsDNA was treated as a chain of rigid monomers, and bending energy was assigned to each angle between adjacent monomers. Thus, the Hamiltonian was the sum of the total bending energy of the polymer from all monomer steps $\sum\limits_{i = 1}^{N - 1} {\frac{k}{2}\theta _{i,i + 1}^2 }$ for WLC and $\sum\limits_{i = 1}^{N - 1} {B\left| {\theta _{i,i + 1} } \right|}$ for linear subelastic chain (LSEC) where $\theta _{i,i + 1}$ is the angle between the i-th monomer and the i+1-th monomer and the harmonic potential $\frac{K}{2}\left( {r - r_0 } \right)^2$ with stiffness *K* that restrains the end-to-end distance near $r_0$. For the WLC model, each base pair was treated as a monomer, similar to the dinucleotide model. On the other hand, the LSEC model assumes that DNA has a linear bending energy function with a monomer length of ∼7bp (21,37). The bending rigidity constants were chosen so that both models predict a persistence length of ∼50 nm in the long limit (see Supplementary Information). We also considered the KWLC model (28) that allows for kink formation at large bending angles. The bending energy for the (i,i+1)-th dinucleotide step is $\min \left( {\frac{1}{2}k\theta _{i,i + 1}^2 ,h + (\theta _{i,i + 1} - b)^6 } \right)$. In this formula, $k$ is the bending rigidity which is the same as in the WLC model and $h$ is the energy barrier for kinking (28). $b$ was fixed to 0.3 radians (if not mentioned otherwise) to allow the kinks to adopt bending angles up to 90°.

Except for the bias potential for umbrella sampling, we did not apply constraints on relative bending or torsional angles between the two ends because flexible gaps at the ends of the linker effectively relax bending and torsional stress. The lack of angular constraints in the loop geometry of our DNA construct is supported by the observation that the J-factor of DNA with gaps does not oscillate with the helical phase of DNA (38), in contrast to intact DNA circles (39).

In principle, the force can be obtained from the derivative of the unbiased radial probability distribution at $r_0$ according to the thermodynamic relation:

(1) \begin{equation*} f(r_0 ) = - k_B T\left. {\frac{{\partial \log P(r)}}{{\partial r}}} \right|_{r_0 }. \end{equation*}

Because short distances are rarely populated, we used Monte Carlo (MC) simulation with umbrella sampling where a biasing harmonic potential of stiffness $K$ is applied near $r_0$ to obtain a sufficient number of looped conformations. The spring constant $K$ for the biasing potential in the case of the WLC model and the LSEC model was set to 8 pN.nm/(1 bp)^2^ and 400 pN.nm/(7 bp)^2^, respectively. The biased force ($f^b$) is then given by (40)

(2) \begin{equation*} f^b (r) = f^u (r) + K(r - r_0 ). \end{equation*}

Thus, the unbiased force ($f^u$) is equal to $f^b$ if evaluated at $r_0$, which enables us to use Equation (2) to calculate $f^u$ directly from a biased radial probability distribution. Since derivatives are sensitive to statistical noise, we instead used an approximation that contains averaging (41)

(3) \begin{equation*} f(r_0 ) = - \frac{{k_B T}}{{{\mathop{\rm var}} (\delta r)}}\left\langle {\delta r} \right\rangle , \end{equation*}

where $\delta r$ is the deviation of the end-to-end distance from $r_0$. $ < \delta r >$ and ${\mathop{\rm var}} \left( {\delta r} \right)$ are the mean and the variance of these deviations, respectively. Pivot moves were used to sample the conformational space of the chain, and Metropolis criterion was applied to accept conformations consistent with the Boltzmann distribution. The chain was equilibrated for 10^5^ MC steps starting from the minimum energy conformation, and ∼10^7^ conformations after equilibration were used to obtain $P\left( r \right)$. The calculated force for a specific loop size did not depend on the value of $K$. For the WLC and the LSEC models with monotonically increasing bending energy, the calculated force varied little between simulations. For the KWLC model with a discontinuous slope, the calculated force for small loop sizes was more variable and, therefore, we increased the number of simulations until the standard error of the mean (SEM) was smaller than 8% of the mean.

### Analysis of linker lifetime versus force

To analyze the linker lifetime versus shear force, we performed linear regression with the ‘robustfit’ function (MATLAB). We also examined how the goodness of fit changes with the range of fitting using the standard regression error or root mean squared error (RMSE) as an indicator.

### J factor calculation

The J factor was calculated by Weighted Histogram Analysis Method (WHAM) (42,43). A number of umbrella sampling simulations were carried out, each having its own restraint energy $U_j \left( {r_k } \right)$, where $j$ is the simulation index and $k$ is the bin index. In the *j*-th simulation, one obtains the number of counts $n_{j,k}$ in the *k*-th bin with the total counts equal to $N_j = \sum\limits_k {n_{j,k} }$. Using the bias factor in each bin $c_{j,k} = \exp ( - U_j (r_k ))$, we can obtain the radial probability density of the unrestrained chain ($p_k^0$)

(4A) \begin{equation*} p_k^0 = \frac{{\sum\limits_j {n_{j,k} } }}{{\sum\limits_j {f_{k,j} N_j } }}, \end{equation*}

(4B) \begin{equation*} f_{k,j} = \frac{{c_{j,k} }}{{\sum\limits_k {c_{j,k} p_k^0 } }}. \end{equation*}

These equations were solved iteratively by updating the equations until $p_k^0$ converges. We adjusted the spring constant and restraint coordinates so that there is significant overlap between adjacent histograms. Typically, each individual histogram was built from 10^6^ chains. The J factor was obtained by normalizing $p_k^0$, dividing it by $4\pi r^2$ and converting it to molar units. This numerical procedure is shown in Supplementary Figure S5.

### Minicircle simulations

The MC simulation for a DNA minicircle was implemented as previously described (44). We applied the KWLC bending energy to each link and calculated the total bending energy of the minicircle. Random conformations generated by crankshaft rotations were selected based on the Metropolis criteria. In one course of simulation, 15 × 10^6^ conformations were typically collected. To enhance the sampling efficiency, we randomly picked angles for the crankshaft rotation from two uniform distributions across two intervals, [−90°, 90°] and [−10°, 10°]. For each accepted conformation, all the dinucleotide angles were recorded to determine if the minicircle has kinks. A kink was assigned if the bending angle exceeds the critical kink angle defined as the intercept of the two energy terms in Equation (6). For each loop size, we calculated the kinking probability, which is the fraction of accepted conformations with at least one kink.

## RESULTS

### Smaller DNA loops are less stable

DNA molecules with sticky ends were constructed using a PCR-based protocol (35,36). Cy3 and Cy5, the donor–acceptor pair for FRET is incorporated near the sticky ends of the DNA so that loop stabilization by the sticky ends results in high FRET efficiency. A biotin linker extends from one end for surface immobilization (Figure 1B). The DNA sequences used in this study are random and do not contain A-tracts which can produce curved molecules. The DNA molecules immobilized to the surface are first stabilized in the looped state in a buffer with 2 M NaCl. Once equilibrium is reached, an imaging buffer containing 50–200 mM NaCl is perfused into the sample chamber, and Cy3 and Cy5 fluorescence intensities are continuously monitored (Figure 1C). The number of remaining high-FRET molecules is recorded as a function of time, and the decay curve is fitted with a single exponential function to extract the linker lifetime (Figure 1D).

**Figure 2.**
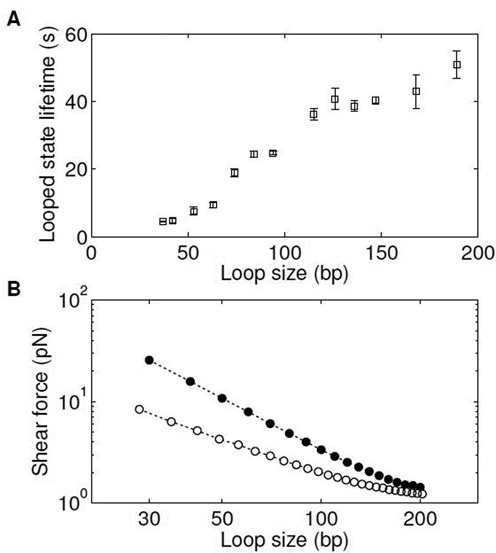
Shear-force-dependent loop breakage. (**A**) Looped state lifetime versus loop size. The loop lifetime at various sizes was measured in 50 mM NaCl. The error bar is the SEM from at least four measurements. (**B**) Line-scatter log–log plot of calculated shear force versus loop size. The shear force is calculated from the biased MC simulation using the WLC model with a persistence length of 50 nm (filled circles) or the LSEC model (open circles). For each loop size, we performed three simulations, each with ∼5 × 10^6^ accepted conformations. The error bar size is typically smaller than the size of the symbol. See Materials and Methods for more information on the simulation details.

We repeated this salt drop experiment for different lengths of DNA molecules ranging from 40 to 200 bp. In this length range, the bending energy dominates the free energy of looping (Supplementary Figure S1A). Since the total bending energy of the loop increases as the loop size decreases (Supplementary Information and Supplementary Figure S1A), we expect smaller loops to become less stable. In support of this notion, the linker lifetime decreased as the DNA length was reduced (Figure 2A).

### Shear force can be calculated from biased MC simulation

To gain more insights into the energetics of strong DNA bending, we formulate the relationship between the lifetime and the loop size by using the shear force exerted on the linker duplex as an intermediate variable. The lifetime ($\tau$) of the linker duplex of length $r_0$ subjected to a shear force ($f$) can be modeled by the Bell relationship (45,46)

(5A) \begin{equation*} \tau (f) = \tau (0){\rm exp}\left( { - \frac{{f\Delta r_0 }}{{k_B T}}} \right), \end{equation*}

(5B) \begin{equation*} \log \tau (f) = \log \tau (0) - \frac{{f\Delta r_0 }}{{k_B T}}, \end{equation*}

where $\Delta r_0$ is the elongation of the linker duplex at the transition state. To obtain the shear force, we considered two continuous polymer models: the WLC model and the LSEC model. The WLC model is the canonical elastic DNA model with a quadratic dependence of deformation energy on bending angle. In comparison, the LSEC model assumes a linear relationship between them and has been proposed as a phenomenological DNA model in the strong bending regime (21,37). The parameters of both models are strongly constrained by the persistence length of ∼50 nm in the long limit. When constrained in this fashion, the LSEC model predicts high-curvature conformations more frequently than the WLC model (21,37). We performed the biased MC simulation to calculate the shear force as a function of loop size (see Materials and Methods). The LSEC model produces a significantly weaker shear force and a more moderate length dependence than the WLC model (Figure 2B). As expected, our calculated WLC shear force (see Supplementary Figure S1A) is in good agreement with the result from the elastic rod approximation below 100 bp (47,48). We also note that the calculated shear force depends only weakly on $r_0$ near the value chosen for our analysis (Supplementary Figure S1B).

### Loop lifetime versus shear force reveals softening transition of a WLC

**Figure 3.**
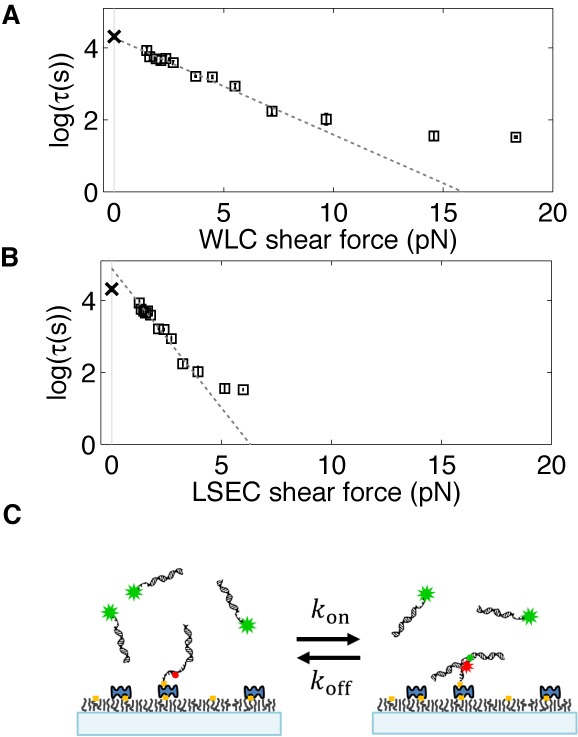
The relationship between the duplex linker lifetime and shear force. The natural logarithm of the lifetime measured in 50 mM NaCl is plotted as a function of shear force calculated from the WLC (**A**) and the LSEC model (**B**). Linear regression yields the zero-force lifetime ($\tau \left( 0 \right)$ and the separation distance ($\Delta r_0$) for duplex dissociation. Only the lifetimes for DNA loops larger than 60 bp are included in the regression. Data for loop size <60 bp are excluded from the linear regression based on the RMSE analysis (see Supplementary Figure S2A). (**C**) The zero-force lifetime ($\tau \left( 0 \right)$) measurement from dissociation kinetics of a linear dimer. In this experiment, the linker formed between the sticky ends of the DNA molecules does not experience a shear force, and therefore, the dissociation lifetime corresponds to $\tau \left( 0 \right)$. DNA molecules are composed of an 18-bp duplex and a 13-mer single-stranded overhang, and are identical to the end segments of the DNA molecule as depicted in Figure 1B. The zero-force lifetimes (marked with ‘x’) averaged from four measurements are plotted in (A) and (B) for comparison with the two models. The error bar for this data point is smaller than the symbol.

We plotted the logarithm of the measured lifetime versus the calculated forces, which is expected to be a straight line according to Equation (5B). As shown in Figure 3, the overall relationship follows a straight line between 60 and 200 bp, but deviates from it at smaller loop sizes (also see the RMSE analysis in Supplementary Figure S2A). This deviation indicates a softening transition of the loop where the actual force becomes weaker than the force predicted by each model. This critical loop size at which the deviation occurs is quite robust, independent of the choice of the persistence length (Supplementary Figure S2B). The relationship in the linear regime can be fitted with Equation (5B) to obtain the negative slope ($\Delta r_0$) and the y-intercept ($\tau (0)$), both of which are related to the dissociation kinetics of the linker duplex. Since the WLC and LSEC models predict markedly different $\Delta r_0$ (1.10 ± 0.14 nm versus 3.18 ± 0.48 nm) and $\tau (0)$ (72.24 ± 10.28 s versus 132.83 ± 6.20 s), we can compare these fitting parameters with experimental values to identify the correct model before the softening transition.

The linker lifetime with zero shear force, $\tau (0)$, can be measured using the same linker without the loop. For this experiment, we prepared two separate DNA molecules identical to the end segments of the DNA used in the unlooping assay so that they can form the same linker without the shear force (Supplementary Figure S3A and Supplementary Information). We immobilized the Cy5 DNA on the surface and introduced the Cy3 DNA at ∼20 nM concentration (Figure 3C). Linker formation and separation resulted in two-state fluctuation in Cy5 intensity due to FRET (Supplementary Figure S3B). Linker separation could be well described by first-order kinetics, from which the lifetime was extracted. We find that the measured $\tau (0)$ (marked with ‘x’ in Figure 3A and B) agrees well with the WLC model prediction, but not with LSEC.

On the other hand, $\Delta r_0$ was previously measured to be 1 Å, per base pair by pulling short DNA duplexes at opposite 5’-ends (49). In our stretched linker duplex, the total number of complementary base pairs is 13, but the largest number of consecutive base pairs is 9 due to the Cy5 in the backbone. Therefore, $\Delta r_0$ can be estimated to be in the range of 0.9–1.3 nm, which includes the prediction of the WLC model but not the LSEC model. Since both parameters $\Delta r_0$ and $\tau (0)$ are compatible with the WLC model, but not with the LSEC model, we conclude that the free energy of dsDNA loop as small as 60 bp is better described by the WLC model.

### Softening transition is sensitive to magnesium

**Figure 4.**
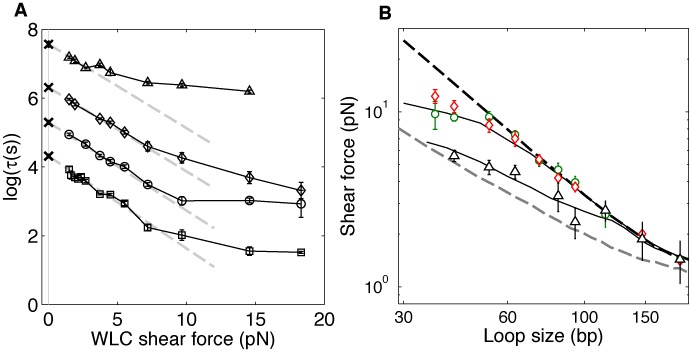
The effect of sodium and magnesium salt on strong dsDNA bending. (**A**) The logarithm of the measured loop lifetime is plotted as a function of the predicted WLC shear force in different [Na^+^]s (squares: 50 mM, circles: 100 mM, diamonds: 200 mM) and in 5 mM [Mg^2+^] (triangles). In sodium buffers, the softening transition appears at ∼8 pN, which corresponds to a loop size of 60 bp whereas in 5 mM [Mg^2+^], it is noticeable at ∼3 pN, which corresponds to a loop size of 100 bp. For the WLC model, we assumed a constant persistence length of 50 nm (16,17). The lifetimes at zero force (‘x’ symbols) were measured from the dimer dissociation experiments. For each salt condition, three separate measurements were performed. Linear fitting of data points in the elastic regime (gray dashed lines) yields almost identical negative slopes independent of [Na^+^] concentration. (**B**) Shear force extracted from the unlooping experiment in 100 mM [Na^+^] (green circles), 200 mM [Na^+^] (red diamonds) and 5 mM [Mg^2+^] (black triangles) are compared with predictions from three DNA models (the axis is in log–log scale). We optimized $h$ and $b$ of the KWLC model to fit the softening transition. The black curves represent the shear forces calculated from the KWLC model. For 50–200 mM [Na^+^], best fit yields $h = 22k_B T$ and $b{\rm } = {\rm }0.3$, and $h = 17k_B T$ and $b{\rm } = {\rm }0.7$ for 5 mM [Mg^2+^]. Also shown are the forces calculated from the WLC model (dotted black curve) and the LSEC model (dotted gray curve). The KWLC model with the lower $h$ is similar to the LSEC model while the KWLC model with the higher $h$ is similar to WLC. Below a certain loop size, the KWLC model predicts a smaller shear force than the WLC model because a kink relieves some of the bending stress.

To confirm that our conclusion is not affected by duplex dissociation kinetics, we conducted the unlooping assay at different [Na^+^] concentrations. As expected, the linker lifetime $\tau (0)$ was prolonged at higher salt concentrations (Figure 4A and Supplementary Figure S3B). Despite changes in loop lifetimes as a function of [Na^+^], all curves exhibit a softening transition near 60 bp, and all $\tau (0)$ values (marked with ‘x’ in Figure 4A) overlap nicely with the values extrapolated by the WLC model. This result further supports our conclusion that the WLC model correctly describes the free energy of dsDNA bending prior to the softening transition.

We also investigated how magnesium affects strong bending of dsDNA. Magnesium is essential for the activity of the ligase in the cyclization assay and the restriction enzyme in DNA minicircle digestion. Therefore, almost all enzyme-based experiments on strong dsDNA bending have been performed in the presence of magnesium at relatively high concentrations (5–10 mM). Interestingly, we found that in the presence of 5 mM [Mg^2+^], the softening transition of dsDNA occurs near 100 bp (Figure 4A). This result indicates that magnesium can dramatically increase the apparent flexibility of dsDNA in the strong bending regime.

## DISCUSSION

Using a FRET-based unlooping assay, we probed the energetics of dsDNA bending in the strong bending regime. We measured the loop lifetime as a function of loop size. In standard sodium concentrations between 50 and 200 mM, the observed relationship in the range between 60 and 200 bp was consistent with the WLC model. Below 60 bp, we observed that dsDNA loses elastic rigidity, which leads to a weaker dependence of the shear force on the loop size. The critical loop size where softening occurs corresponds to a maximum bending angle of 7°/bp in a teardrop shape. In the presence of 5 mM [Mg^2+^], the critical loop size increased to 100 bp, corresponding to 4°/bp. This result suggests that in cyclization experiments that typically use 10 mM [Mg^2+^], subelastic bending can enhance the looping probability of dsDNA shorter than 100 bp. The interpretation of our results relies on the Bell relationship between duplex lifetime and stretching force (45). In general, a bond can dissociate through several different pathways (50), which may give rise to a nontrivial relationship between bond lifetime and the applied force (51). However, our assumption of the Bell model is justified by previous experimental studies (49,52). Notably, a DNA duplex pulled at the opposite 5′-ends by AFM, in the same shear geometry as in our DNA loop, exhibited strand separation kinetics consistent with a single energy barrier along the mechanical separation path. Also, the Chemla group recently demonstrated that DNA duplex dissociation under a constant tensile force follows the Bell relationship by combining fluorescence with optical tweezers (52). In that study, the relationship between $\Delta r_0$ and duplex length ($L$) was extracted to be $\Delta r_0 = {\rm }0.096 \times L$ (nm), and has been more precisely determined as $\Delta r_0 = 0.256 \times \left( {L{\rm } - {\rm }6} \right)$ (nm) (personal communication with Dr Chemla). Either estimation puts $\Delta r_0$ to be in the range consistent with the WLC model but not with the LSEC model.

We showed that continuous polymer models with a single flexibility parameter are not sufficient to explain the observed loop lifetime versus loop size relationship. It was pointed out that finite end-to-end distance of a loop can give rise to a non-monotonic dependence of looping probability on loop size (53). But such geometric effect emerges only when the loop size is comparable to the end-to-end distance. Even with this geometric consideration, the shear force always changes monotonically with loop size (Figure 2B). Therefore, the observed deviation in loop lifetime versus loop size relationship cannot be due to this effect.

The breakdown of continuous models below the critical loop size is likely due to structural transition in the dsDNA helix such as kink formation that was originally proposed by Crick and Klug (54). For free DNA, kinks are rare, transient deformations only occurring at a rate of 10^−4^–10^−5^ (55,56), but they can become significant in sharply bent DNA (57–59). Kinks are expected to have a significantly reduced bending rigidity, but our data indicates that the kink rigidity is not zero because, otherwise, the loop lifetime just below the critical loop size would be similar to $\tau (0)$. Therefore, we rule out the KWLC model with completely flexible kinks (60). Instead we assume kinks to possess non-zero rigidity according to the KWLC model suggested by Vologodskii and Frank-Kamenetskii (28). In this model, the dinucleotide bending energy ($E$) is given by

(6) \begin{equation*} E = \min \left( {\frac{1}{2}k\theta ^2 ,h + (\theta - b)^6 } \right) \end{equation*}

where $k$ is the bending rigidity identical to that of the WLC model, $h$ is the energy barrier of kinking and $b$ specifies the range of bending angles at the kink. This model is conceptually similar to the meltable WLC model (61–63).

To set the lower limit on the energy barrier for kink formation, we varied $h$ while fixing $b$ in our simulation to find $h$ that is most compatible with the observed critical length of 60 bp. The parameter $b$ was chosen to be 0.3 which allows kink angles up to 90° (28) based on other calculations and molecular dynamics simulations (54,57,64). As shown in Figure 4B, $h = 22k_B T$ and $b = 0.3$ can produce a transition in the shear force below 60 bp, which is consistent with our observation. Using this $h$ value, we can also calculate the free energy of kink formation ($\Delta G_k$) (more details in the Supplementary Information) to be $\Delta G_k \approx 18k_B T$, which is similar to the upper limits of previous estimations (44,59). In comparison to $h = 22k_B T$ and $b = 0.3$, the lifetime versus loop size relationship taken at 5 mM [Mg^2+^] yields $h = 17k_B T$ and $b = 0.7$. These parameters correspond to larger kink angles up to 110° and a lower free energy of kink formation of $\Delta G_k = 12k_B T$ which is in good agreement with the thermodynamics of DNA melting (63).

Using the parameters, $h$ and $b$, constrained by our data, we can also determine the probability of kink formation in a DNA minicircle as a function of loop size. We performed a restrained MC simulation of DNA minicircles of various sizes (see Materials and Methods) to measure the frequency of large-angle deflections in thermal equilibrium. In our simulation, we only consider the effect of bending stress on kink formation. As shown in Supplementary Figure S4, in the absence of magnesium, kink formation is negligible even in 60-bp loops due to a high energy barrier. In the presence of 5 mM [Mg^2+^], however, the kinking probability increases sharply with decreasing loop size, approaching unity at 70 bp while remaining insignificant for DNA over 100 bp. This simulation result agrees well with a previous minicircle digestion study that detected kinks in 60-bp minicircles due to bending stress alone (65).

**Figure 5.**
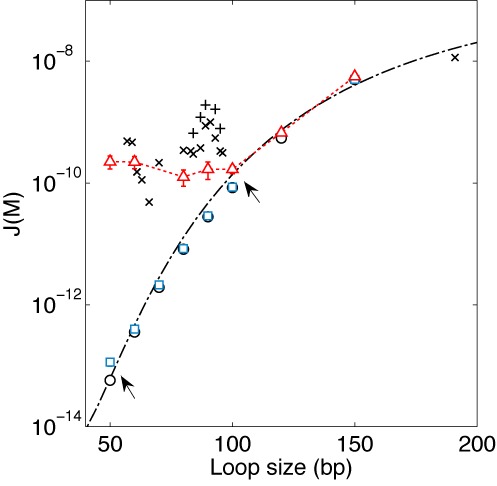
J factor comparison. The J factor was computed using a fixed end-to-end distance of 5 nm without end-to-end angular or torsional constraints. We used the WHAM to calculate J factors predicted by the WLC model (black circles), the KWLC model with $h = 17k_B T$ and $b{\rm } = {\rm }0.7$ (red triangles), and with $h = 22k_B T$ and $b{\rm } = {\rm }0.3$ (blue squares). The dash-dotted line is the WLC prediction according to the Douarche–Cocco approximation (47). For comparison, the J factors from Vafabakhsh and Ha (24) are also shown. Symbols marked with ‘x’ indicate J factors measured from surface-immobilized DNA in 1 M [Na^+^] and symbols marked with ‘+’ from vesicle-encapsulated DNA at 10 mM [Mg^2+^]. For consistency with our calculation, we shifted the original data (24) by 10 bp to the left to account for the linker length. The arrows indicate where the KWLC model deviates from the WLC model. We performed four simulations to generate the SEM error bars for each J factor.

Our results suggest that magnesium can promote subelastic bending above a critical bending angle of 4°/bp by stabilizing large-angle deformations. This interpretation is similar to the conclusion of a recent study with DNA vises (59). Therefore, we considered whether magnesium-facilitated softening could explain high J factors reported previously (24). We thus calculated the J factor as a function of length using the KWLC model with $h = 17k_B T$ and $b = 0.7$ constrained by the data taken at 5 mM [Mg^2+^]. As shown in Figure 5, the KWLC model produces J factors similar to the WLC model prediction above 100 bp as previously demonstrated (22) and also predicts substantially higher J factors for DNA below 100 bp, matching the J factors determined from the single-molecule FRET cyclization study (24) within a factor of 10. The agreement between our KWLC model and the result of the FRET study may be closer if the difference in the buffer condition (5 mM versus 10 mM [Mg^2+^]) and the uncertainty associated with J factor determination is accounted for (Supplementary Information). In the absence of magnesium, however, our KWLC model predicts that the WLC model will be valid at least down to 55 bp (blue squares, Figure 5). This may explain why some studies lacking magnesium did not observe enhanced dsDNA flexibility at short length scales (66,67).

Our unlooping assay enables investigation of strong dsDNA bending in buffer conditions not compatible with the ligation-based cyclization, the FRET-based cyclization and the AFM assay. In the ligation assay, magnesium must be present at high concentrations for ligase activity. For AFM, magnesium is necessary to bind DNA to the surface (68). In the FRET-based cyclization assay, high magnesium or sodium concentration is necessary to produce a statistically significant number of looping events. In this study, we demonstrated that effects of small amounts of monovalent and divalent ions on the elastic limit of dsDNA can be studied separately. Moreover, the unlooping assay is more well suited to the study of kink formation than the cyclization assay because the probability of kink formation increases with bending stress. Our unlooping assay is similar in some ways to previous methods employing small DNA loops (59,69,70). In these studies, electrophoretic mobility or intramolecular FRET of these loops was measured to investigate kinking. Our approach differs from theirs in two ways. First, we measure kinetic decay of the looped state instead of equilibrium distribution between alternative conformations in the looped state. Second, we do not need to include stretching or twisting energy in the Hamiltonian for single-stranded parts or twisted dsDNA. Therefore, our method allows a more direct link between the measurable quantities and dsDNA bending rigidity and holds great promise for studying the effect of sequence, salt and temperature on strong dsDNA bending.

## SUPPLEMENTARY DATA

Supplementary Data are available at NAR Online.

SUPPLEMENTARY DATA
