# Supplementary material for: Probing the elastic limit of DNA bending
Source: Nucleic Acids Res. 2014 Aug 13;42(16):10786–94. doi: 10.1093/nar/gku735 (PMC4176374; doi:10.1093/nar/gku735)
Supplement: SUPPLEMENTARY DATA [file supp_42_16_10786__index.html]

Probing the elastic limit of DNA bending — SUPPLEMENTARY DATA 

# Probing the elastic limit of DNA bending

## SUPPLEMENTARY DATA

**Files in this Data Supplement:**

- SUPPLEMENTARY DATA
