# Supplementary material for: Probing the elastic limit of DNA bending
Source: Nucleic Acids Res. 2014 Aug 13;42(16):10786–94. doi: 10.1093/nar/gku735 (PMC4176374; doi:10.1093/nar/gku735)
Supplement: SUPPLEMENTARY DATA [file supp_gku735_nar-01637-f-2014-File007.pdf]

Supporting Information

Probing the elastic limit of DNA bending

Tung T. Le and Harold D. Kim

School of Physics, Georgia Institute of Technology

837 State Street, Atlanta, GA 30332

DNA sequences (from 5′ → 3′)

Master 210 bp DNA

gtgccagcaacagatagcctatccatagactattacctacaagcccaatagcgtacgggatcatccccgccagttacgtctgccacccttcttaacg acacgtgaagggacgaaccgcatacttacgatcaggcatagatcttacaccgtagcaggtagtgccaggcatcgtgttcgtaaccttacttcaacca ttcgagctcgttgttg

189 bp

gtgccagcaacagatagcctatccatagactattacctacaagcccaatagcgtacgggatcatccccgccagttacgtctgccacccttcttaacg acacgtgaagggacgaaccgcatacttacgatcaggcatagatcttacaccgtagcaggtagtgccaggcatcgcattcgagctcgttgttg

168 bp

gtgccagcaacagatagcctatccatagactattacctacaagcccaatagcgtacgggatcatccccgccagttacgtctgccacccttcttaacg acacgtgaagggacgaaccgcatacttacgatcaggcatagatcttacaccgtcattcgagctcgttgttg

147 bp

gtgccagcaacagatagcctatccatagactattacctacaagcccaatagcgtacgggatcatccccgccagttacgtctgccacccttcttaacg acacgtgaagggacgaaccgcatacttacgatcattcgagctcgttgttg

136 bp

gtgccagcaacagatagcctatccatagactattacctacaagcccaatagcgtacgggatcatccccgccagttacgtctgccacccttcttaacg acacgtgaagggacgaaccgccattcgagctcgttgttg

126 bp

gtgccagcaacagatagcctatccatagactattacctacaagcccaatagcgtacgggatcatccccgccagttacgtctgccacccttcttaacg acacgtgaaggcattcgagctcgttgttg

115 bp

gtgccagcaacagatagcctatccatagactattacctacaagcccaatagcgtacgggatcatccccgccagttacgtctgccacccttcttaacgc attcgagctcgttgttg

94 bp

gtgccagcaacagatagcctatccatagactattacctacaagcccaatagcgtacgggatcatccccgccagttacattcgagctcgttgttg

84 bp

gtgccagcaacagatagcctatccatagactattacctacaagcccaatagcgtacgggatcatcccattcgagctcgttgttg

74 bp

gtgccagcaacagatagcctatccatagactattacctacaagcccaatagcgtaccattcgagctcgttgttg

63 bp

gtgccagcaacagatagcctatccatagactattacctacaagcccattcgagctcgttgttg

53 bp

gtgccagcaacagatagcctatccatagactattacattcgagctcgttgttg

42 bp

gtgccagcaacagatagcctatcccattcgagctcgttgttg

37 bp

gtgccagcaacagatagcccattcgagctcgttgttg

Preparing partially hybridized DNA molecules for measuring the linker lifetime with zero force (italic: double-stranded region)

Cy3-DNA:

5′ - Cy3-ggtaaattcactat *caacaacgagctcgaatg* - 3′ (primer 1)

3′ - *gttgttgctcgagcttac* - 5′ (blocking oligo)

Cy5-DNA:

5′ – BiotinTEG - gaaacatag/ iCy5 /gaatttacc *gtgccagcaacagatagc* -3′ (primer 2)

3′ - *cacggtcgttgtctatcg* - 5′ (blocking oligo)

We mixed equal amounts of the two partially hybridized DNA molecules in annealing buffer (100mM NaCl, 10 mM Tris.HCl pH 7.0, 1 mM EDTA) to obtain a final concentration of 5 μM. The mixture was heated at 95 ºC for 5 min, slowly cooled down to room temperature, and loaded on a polyacrylamide gel (19:1 Acryl:Bis, 15% (w/v) in TBE 1X pH 8.0). Linear dimers were extracted from the gel using an

electroelution kit (G-CAPSULE, 786-001, G-Biosciences) after running the gel at 10 V/cm for ~1 hour (see Supplementary Figure S3A).

Shear force vs. loop length

Here, we derive an approximate relationship between the total bending energy of a circular loop and loop length (L). From this relationship, we can obtain the shear force. We assume that the loop takes the shape of a circular arc with the two ends separated by distance r . If the bending rigidity of the chain is k , the total bending energy of the loop is calculated as

$$E(r)=\frac{k}{2}\int_0^L ds\frac{1}{R(s)^n}\approx\frac{k}{2}\int_0^L ds\frac{1}{\left(\frac{r+L}{2\pi}\right)^n}=2\pi^2 k\frac{L}{(r+L)^n}, \qquad \textbf{(S1)}$$

where s is the distance coordinate along the contour, and R is the radius curvature, which is constant for a circular arc. n is 1 for the linear subelastic chain model, and 2 for the worm-like chain model. We made the assumption that r is much smaller than L . Bending rigidity k is equal to $L_p k_B T$ in the worm-like chain model. Differentiating the bending energy with r , we can obtain the shear force acting along $\vec{r}$,

$$f=-2\pi^2 nk\frac{L}{(r+L)^{n+1}}. \qquad \textbf{(S2)}$$

Thus, at short end-to-end distance, we expect the shear force to scale as L^{-2} for a worm-like chain, and L^{-1} for a subelastic chain. As shown in Supplementary Figure S1A, this approximate expression can explain the scaling of force vs. length computed from the Monte Carlo (MC) simulation to some degree. However, it overestimates the absolute force values because the dominant loop conformation of a worm-like chain is closer to a teardrop, which is overall less stressed than a circular arc.

A more accurate description of the shear force requires the full probability distribution of end-to-end distances. An exact analytical expression does not exist in a closed form, and therefore, we use an approximation that best describes the probability distribution at short end-to-end distances in the stiff limit(1). Douarche and Cocco proposed such approximation (DC approximation) that considers both the Boltzmann weight due to the elastic energy of the loop and fluctuation around the minimum energy conformation(2, 3). The cyclization factor is given by(3)

$$j(L,r)=\frac{1.66\times 112.04}{L_P{}^3}\left(\frac{L+2r}{L_P}\right)^{-5} e^{0.246\frac{L+2r}{L_P}} e^{-14.055\frac{L_P}{L+2r}}. \qquad \textbf{(S3)}$$

Multiplying this by $4\pi r^2$ and differentiating, we have

$$f = -k_B T\left(\frac{2}{r} - \frac{10}{L+2r} + \frac{0.492}{L_P} + \frac{28.11 L_P}{L^2}\right). \quad \text{(S4)}$$

Using $L_p = 50\text{nm}$ and $r = 5\text{nm}$ (14.7 bp), we obtain the relationship between the shear force and DNA length in units of base pair number (N_{bp})

$$f\,[\text{pN}] \approx -\frac{49849}{N_{bp}{}^2} - 1.68 + \frac{120.5882}{N_{bp} + 29.4}. \quad \text{(S5)}$$

This expression with no further adjustment can well describe the scaling of the relationship, but overestimates the force almost by a constant scaling factor. If we multiply the force by 0.6, we find an excellent agreement across the length range of interest. It is not surprising that DC approximation overestimates the absolute force value. When compared with the exact density, DC probability density is shown to have a steeper slope at short extension(1), which results in slightly higher force values.

For loops with the contour length shorter than the persistence length, the loop free energy is expected to be dominated by bending energy. We thus used the elastic rod approximation to calculate the shear force, ignoring the entropic contribution. The total bending energy ($E_{WLC} = \sum_{i=1}^{N-1} \frac{k}{2} \theta_{i,i+1}^2$) of the minimum energy conformation was calculated as a function of end-to-end distance through either elliptic integrals(2, 4) or the constrained nonlinear optimization in MATLAB. To obtain the shear force, the total bending energy was numerically differentiated near r_0. As shown in Supplementary Figure S1A, the shear forces due to the bending energy alone agree well with the numerical values from MC simulation for DNA less than 100 bp, which further confirms the validity of our MC simulation.

Parameter choice for polymer models

The length of the monomer and the value of the rigidity constant are chosen so that the known statistical mechanical properties of the polymer can be reproduced by simulation. In the case of dsDNA, these parameters can be determined based on the persistence length (L_P) of the polymer, which is approximately 50 nm. A linear dsDNA molecule longer than the persistence length can be well described as a worm-like chain, and the mean-square end-to-end distance $\langle R^2 \rangle$ is related to its contour length *L* as

$$\left\langle R^2 \right\rangle = 2L_P L\left[1-\frac{L_P}{L}\left(1-e^{-L/L_P}\right)\right]. \quad \textbf{(S6)}$$

To calculate the mean-square end-to-end distance $\left\langle R^2 \right\rangle$, one can generate a large set of chains using the Gaussian sampling method. For the WLC model, we chose the bending rigidity constant k to be $73.53\ k_B T$ for each 1-bp long monomer. For the LSEC model(5, 6), we chose $B =\ 7.84 k_B T$ for each 7-bp long monomer (2.37 nm). The chosen parameters all predict a persistence length of ~50 nm at large length scales.

J factor

The looping probability (P_1) is the colocalization probability of two reactive ends of the same polymer within a small reaction volume δV. We do not know *a priori* what δV is, but it should be small enough to allow for the two ends of the polymer to react. Therefore, for cyclization of dsDNA with complementary single-stranded overhangs, its dimension should be on the order of the length of the single-stranded overhang (~5 nm). The J factor is the effective concentration of one freely diffusing reactive end around the other that would give rise to the same colocalization probability, and can be determined without knowledge of δV.

Without losing generality, we can fix one reactive end inside δV and let other reactive ends freely diffuse at a molar concentration of $\left[X\right]$. The rate of a reactant diffusing into δV is proportional to $\left[X\right]$ ($k_{in}\left[X\right]$) whereas the rate of the reactant diffusing out of the volume is concentration-independent (k_{out}). In typical aqueous reactions, the diffusive encounter between the two ends is much slower than the diffusive separation ($k_{out} \gg k_{in}[X]$) (7). The equilibrium probability of intermolecular colocalization (P_2) is a function of $\left[X\right]$:

$$P_2([X]) = \frac{k_{in}[X]}{k_{in}[X]+k_{out}} \approx \frac{k_{in}}{k_{out}}[X]. \quad \textbf{(S7)}$$

Therefore, the J factor is defined by

$$P_1 = P_2(J) \ \therefore J = P_1 \frac{k_{out}}{k_{in}}. \quad \textbf{(S8)}$$

The J factor can be determined by measuring both intramolecular and intermolecular reaction kinetics. Both reactions follow a three-state reaction kinetics scheme:

$$a \rightleftharpoons b \rightarrow c \qquad \textbf{(S9)}$$

Here, b is the state of end-to-end colocalization without interaction, and c is the high-FRET state stabilized by end-to-end annealing. If $k_{b\to c} \ll k_{b\to a}$, the apparent rate of c formation (k_c) is proportional to the equilibrium probability of state b:

$$k_c \approx \frac{k_{a\to b}}{k_{a\to b} + k_{b\to a}} k_{b\to c} = P_b k_{b\to c}. \qquad \textbf{(S10)}$$

We denote the rate of annealing ($k_{b\to c}$) as f_1 for looping and f_2 for dimerization. The apparent looping rate (k_1) is

$$k_1 \approx P_1 f_1, \qquad \textbf{(S11)}$$

and the apparent dimerization rate (k_2) is

$$k_2 \approx P_2 f_2 \approx \frac{k_{in}}{k_{out}}[X] f_2, \qquad \textbf{(S12)}$$

where we used Equation S7. The second-order rate constant $k_2/[X]$ is usually referred to as the annealing rate constant in most other studies(8–10). According to Equation S8, the J factor is related to the apparent rates by

$$J = \frac{k_1}{k_2/[X]} \cdot \frac{f_2}{f_1}. \qquad \textbf{(S13)}$$

Therefore, only if $f_1 = f_2$ can we determine the J factor from the apparent rates in an unbiased manner.

For looping of long dsDNA, $f_1 = f_2$ is generally accepted(7). For looping of short dsDNA, however, $f_1 = f_2$ may be violated. In dimerization, the two ends approach each other from all 4π steradians. In many of these colocalization events, the sticky ends are not optimally aligned for annealing. In looping, the reactive ends approach each other at a much narrower range of angles. As a result, the dangling overhangs with intrastrand stacking(11) may find each other in an anti-parallel orientation more often than in free diffusion. Hence, the entropic barrier for f_1 would be lower than for f_2. This effect is conceptually similar to rate enhancement in intramolecular reactions that far exceeds local concentration effect due to entropy(12) or orientation-dependent reactivity(13, 14).

Calculating the free energy of kink formation

We adopted the computational method in(15), which is also conceptually similar to a more theoretical approach(16). We considered the dinucleotide bending energy $E(\theta)$ with both the elastic bending term and kinking term using the functional form in Equation 6. The critical kink angle (β) was defined as the intercept of the two terms. The equilibrium probability density ($p(\theta)$) or the partition function of the bending angle θ is proportional to the multiplicity of $sin(\theta)$ and the Boltzmann factor

$$p(\theta) = \sin(\theta)\exp\left(-E(\theta)/k_B T\right). \quad \textbf{(S14)}$$

The kinking probability (P_k) is the probability for θ to exceed the critical kink angle β, which is

$$P_k = \frac{\int_{\beta}^{\pi} \sin(\theta)\exp\left(-E(\theta)/k_B T\right) d\theta}{\int_{0}^{\pi} \sin(\theta)\exp\left(-E(\theta)/k_B T\right) d\theta}. \quad \textbf{(S15)}$$

The free energy of kink formation ΔG_k can be directly calculated from P_k as $\Delta G_k = -k_B T \log\left(P_k\right)$. For example, if we consider an energy function $h + (\theta - b)^6$ with $h = 15 k_B T$ and $b = 0.3$ for kink formation, $\Delta G_k = 10.6 k_B T$. If we assume no additional energy cost of kinking(17), we have a little lower ΔG_k of $9.4 k_B T$, as expected.

Supplementary figure S1. Shear force calculation. **(A)** The shear force vs. loop size. The symbols are obtained from the MC simulation. Solid and hollow squares are for WLC and LSEC, respectively. The relationship is plotted on log-log axes to highlight the scaling. Douarche and Cocco (DC) approximation with no adjustment (purple dotted curve) and with scaling by a factor of 0.6 (red dashed-dotted curve) are also shown. The elastic rod approximation was also used to calculate the shear force by using either elliptic integrals (black solid curve)(2, 3) or the constrained nonlinear optimization in MATLAB (thick blue curve). All forces were evaluated at 5-nm end-to-end distance. **(B)** Shear force vs. end-to-end distance. The mean shear force was calculated from the WLC model for different loop sizes and different end-to-end distances (r_0). The shear forces at different loop sizes (square: 40 bp, circle: 70 bp, diamond: 100 bp, triangle: 130 bp) decrease only slightly as a function of the end-to-end distance. Since the linker duplex is extended by ~1 nm before dissociation, our estimated force can be variable by ~5% for all loop sizes tested.

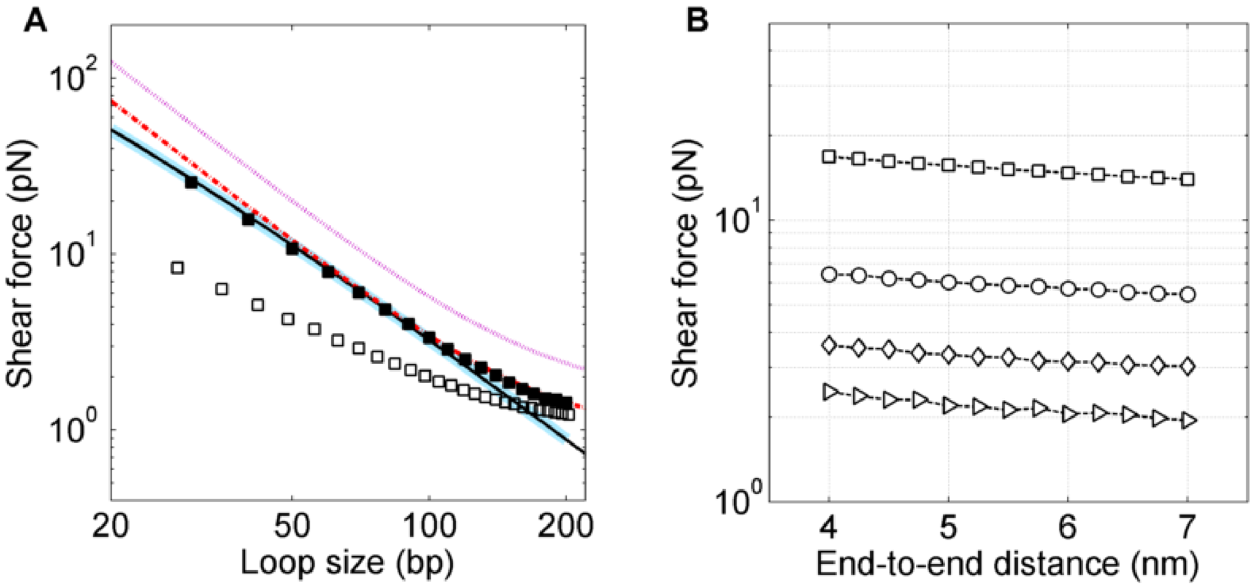

Supplementary figure S2. Deviation of the measured loop lifetime from model predictions. **(A)** RMSE analysis. The linear regression was performed with the 'robustfit' function (MATLAB) on the logarithm of loop lifetime vs. shear force. To identify outliers, we compared the RMSE (root mean squared error) values resulting from different ranges of fitting. For example, the last point is obtained when the entire range of 13 loop sizes from 189 bp down to the smallest 37 bp were included in the fitting. Including the last few points significantly increases the regression error, which indicates that the linear relationship predicted by Equation 4 no longer holds for loop sizes smaller than 60 bp. Thus, we did not include three points corresponding to 37, 42, and 53 bp in the regression when extracting the fitting parameters, $\tau(0)$ and Δr_0. **(B)** Dependence of the critical loop size on persistence length. The logarithm of the loop lifetime is plotted against shear forces (f_{WLC}) calculated with 45-nm (black) and 55-nm (red) persistence lengths. The dashed lines are the linear regressions from the RMSE analysis. Different persistence lengths yield a similar critical loop size where the measured loop lifetime deviates significantly from the WLC model prediction.

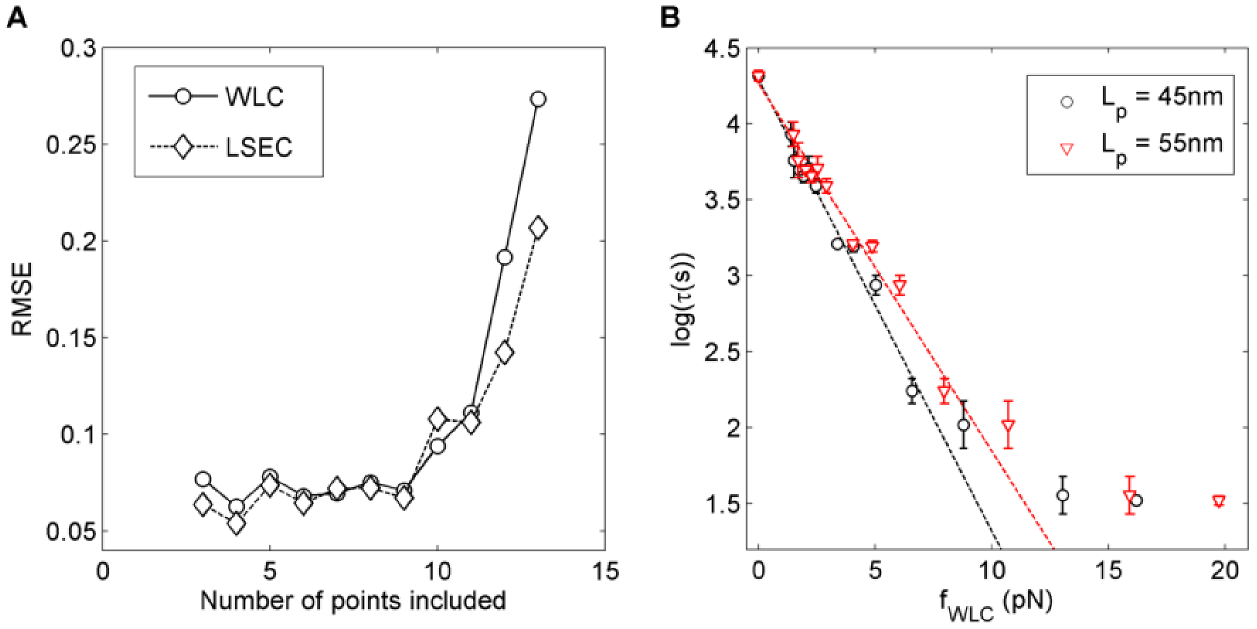

Supplementary figure S3. $\tau(0)$ measurement. **(A)** Polyacrylamide gel image of the hybridized oligos. From left to right, primer 1 only, 1:1 mixture of primer 1 and its blocking oligo, primer 2 only, Lane 4: 1:1 mixture of primer 2 and its blocking oligo. **(B)** Typical time traces of reversible linker formation and separation in 50, 100 and 200 mM [Na^+] (from top to bottom). Linker formation results in a burst in Cy5 intensity due to FRET. The survival probability of the dimer since t = 0 is fitted with a single exponential function to extract the linker lifetime at zero force $\tau(0)$. The concentration of the free monomer was adjusted to obtain similar binding rates at different [Na^+].

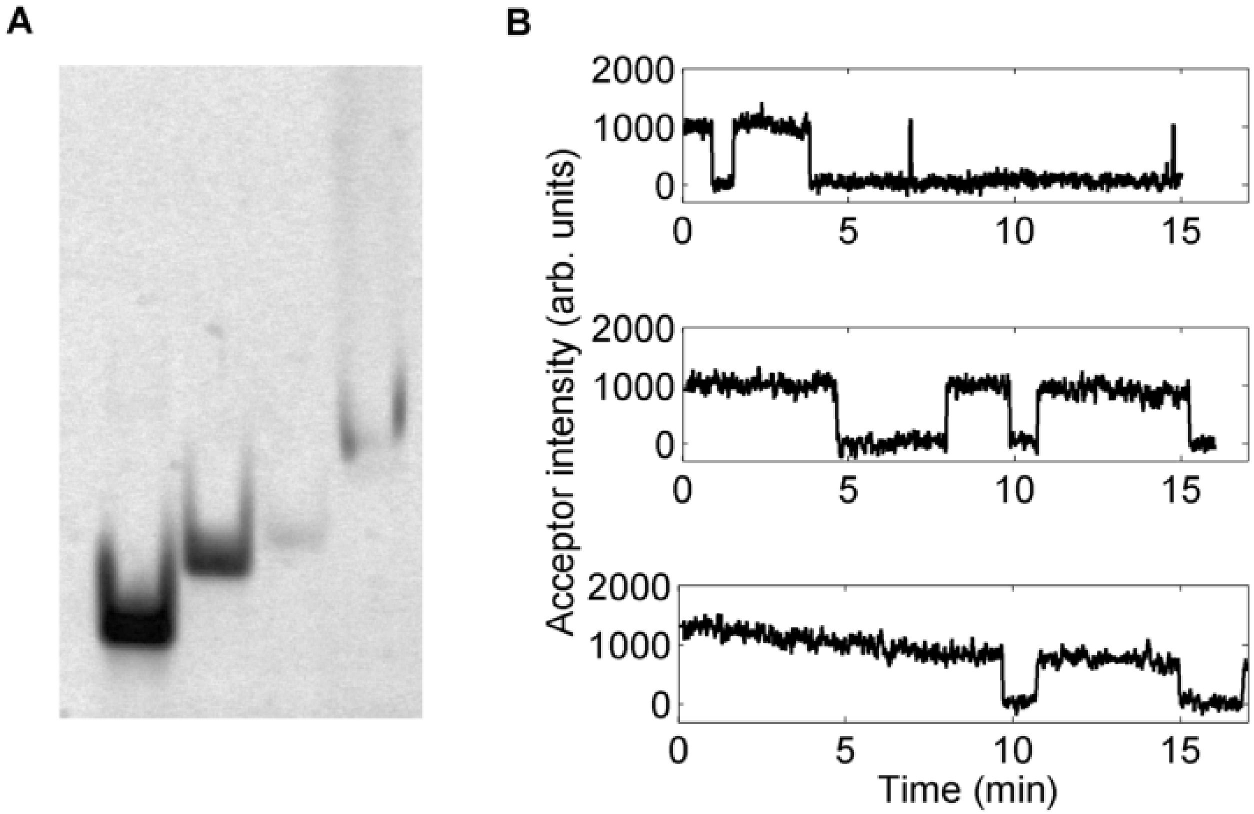

Supplementary figure S4. Kinking probability in DNA minicircles. The kinking probabilities of DNA minicircles were calculated as a function of loop size using the KWLC model with different free energies of kink formation (squares: $\Delta G_k = 18k_BT$ ($h = 22k_BT$, $b = 0.3$), circles: $\Delta G_k = 12k_BT$ ($h = 17k_BT$, $b = 0.7$). The SEM error bar for each loop size was calculated from 5 simulations.

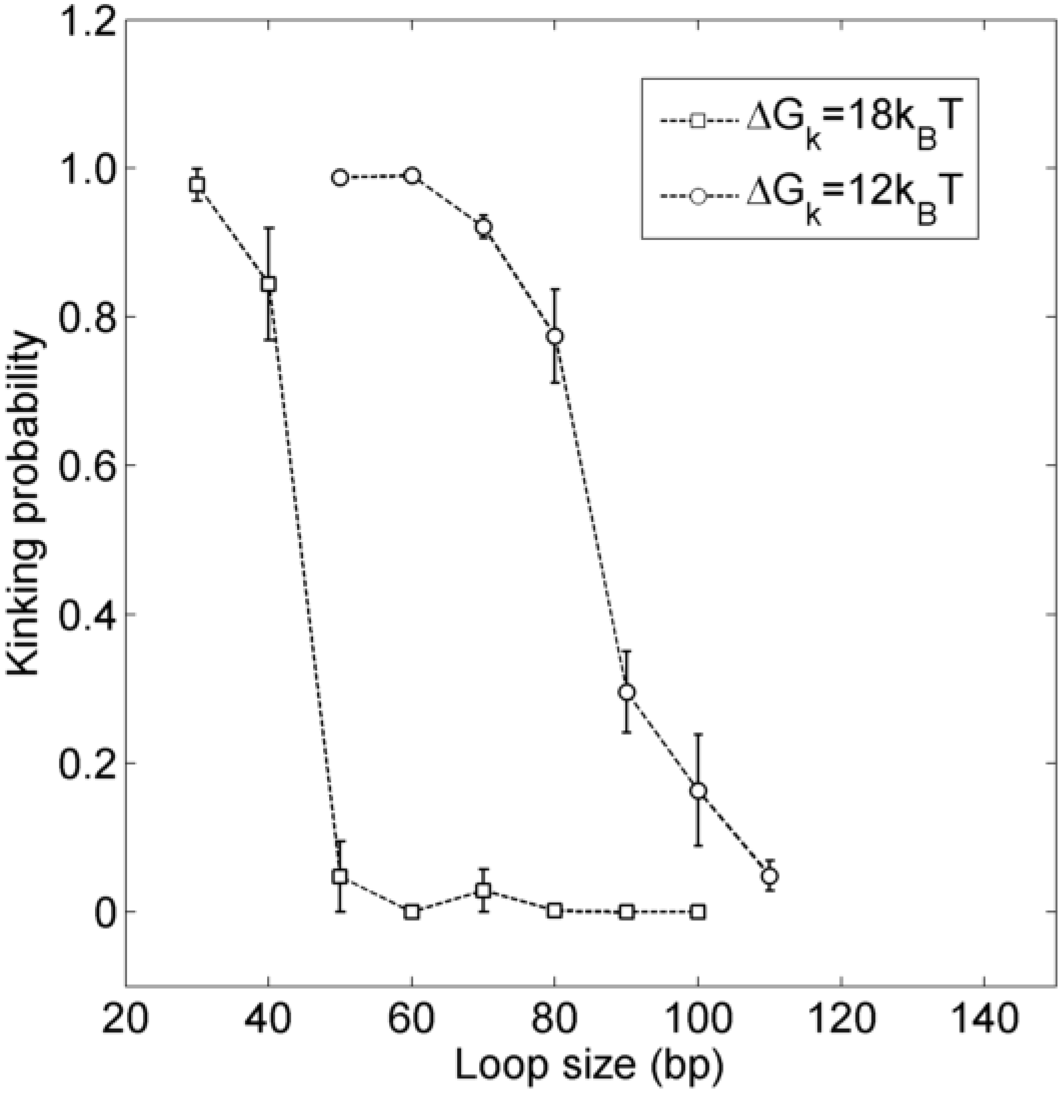

Supplementary figure S5. J factor calculation by the weighted histogram analysis method. **(A)** Umbrella sampling was performed at every 10-bp step. The spring constant was chosen so that neighboring histograms overlap significantly. Each histogram was obtained from 10^6 MC conformations after 100,000 thermalization steps. **(B)** The radial probability distribution was obtained by iterating through Equation 4. The J factor in nanomolar units can be obtained by dividing the amplitude of the radial probability distribution by $4\pi r^2 \Delta r$ and multiplying by 4.24×10^{10}.

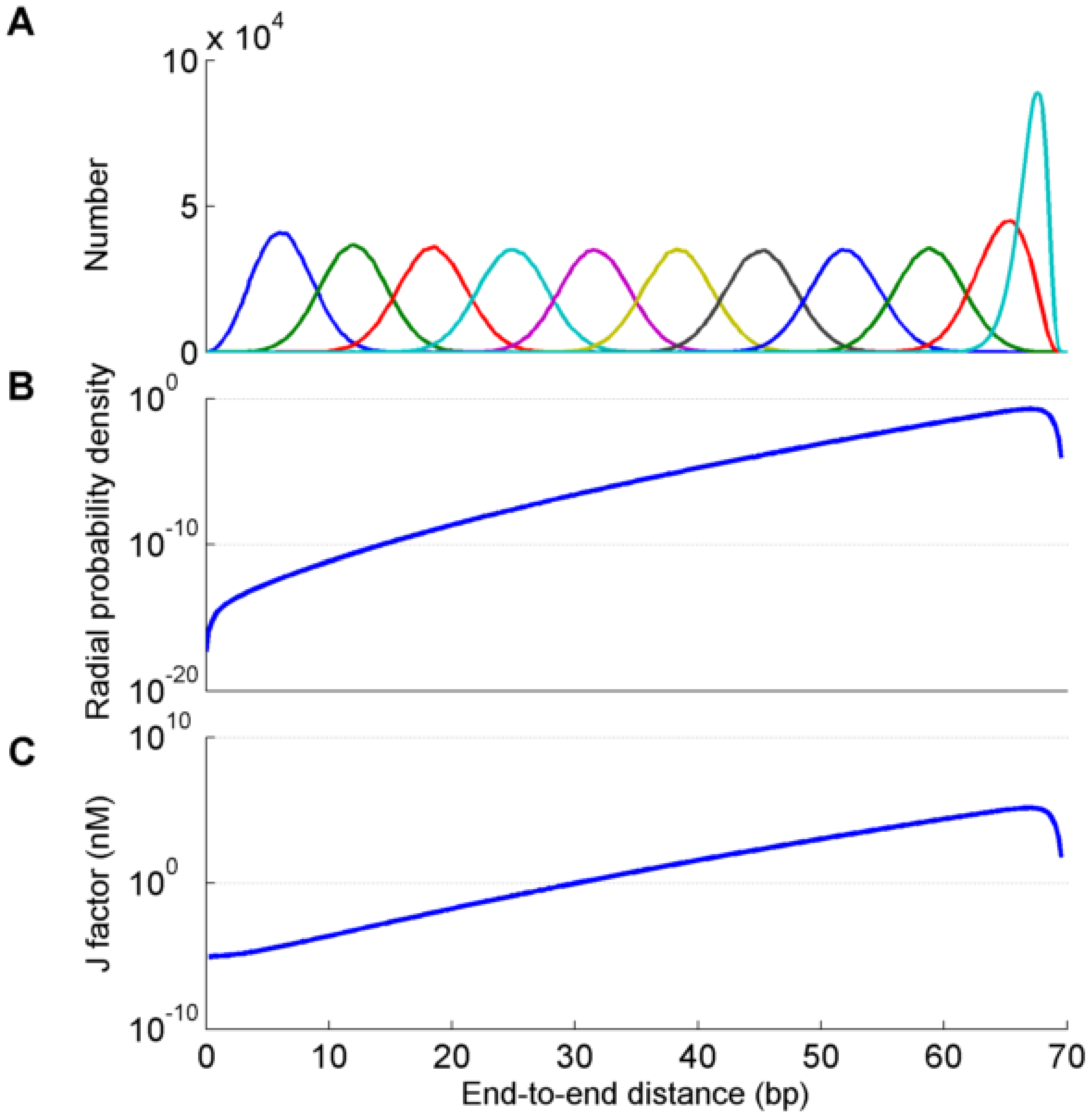

Supplementary References

1. Becker, N.B., Rosa, A. and Everaers, R. (2010) The radial distribution function of worm-like chains. *Eur. Phys. J. E*, **32**, 53–69.

2. Douarche, N. and Cocco, S. (2005) Protein-mediated DNA loops: Effects of protein bridge size and kinks. *Phys. Rev. E*, **72**, 061902.

3. Allemand, J.-F., Cocco, S., Douarche, N. and Lia, G. (2006) Loops in DNA: An overview of experimental and theoretical approaches. *Eur. Phys. J. E*, **19**, 293–302.

4. Purohit, P.K. and Nelson, P.C. (2006) Effect of supercoiling on formation of protein-mediated DNA loops. *Phys. Rev. E*, **74**, 061907.

5. Wiggins, P.A., van der Heijden, T., Moreno-Herrero, F., Spakowitz, A., Phillips, R., Widom, J., Dekker, C. and Nelson, P.C. (2006) High flexibility of DNA on short length scales probed by atomic force microscopy. *Nat Nano*, **1**, 137–141.

6. Wiggins, P.A. and Nelson, P.C. (2006) Generalized theory of semiflexible polymers. *Phys. Rev. E*, **73**, 031906.

7. Wang, J.C. and Davidson, N. (1966) On the probability of ring closure of lambda DNA. *J. Mol. Biol.*, **19**, 469–482.

8. Gao, Y., Wolf, L.K. and Georgiadis, R.M. (2006) Secondary structure effects on DNA hybridization kinetics: a solution versus surface comparison. *Nucleic Acids Res.*, **34**, 3370–3377.

9. Cisse, I.I., Kim, H. and Ha, T. (2012) A rule of seven in Watson-Crick base-pairing of mismatched sequences. *Nat. Struct. Mol. Biol.*, **19**, 623–627.

10. Rauzan, B., McMichael, E., Cave, R., Sevcik, L.R., Ostrosky, K., Whitman, E., Stegemann, R., Sinclair, A.L., Serra, M.J. and Deckert, A.A. (2013) Kinetics and Thermodynamics of DNA, RNA, and Hybrid Duplex Formation. *Biochemistry (Mosc.)*, **52**, 765–772.

11. Ohmichi, T., Nakano, S., Miyoshi, D. and Sugimoto, N. (2002) Long RNA Dangling End Has Large Energetic Contribution to Duplex Stability. *J. Am. Chem. Soc.*, **124**, 10367–10372.

12. Page, M.I. and Jencks, W.P. (1971) Entropic Contributions to Rate Accelerations in Enzymic and Intramolecular Reactions and the Chelate Effect. *Proc. Natl. Acad. Sci.*, **68**, 1678–1683.

13. Dafforn, G.A. and Koshland Jr., D.E. (1971) The sensitivity of intramolecular reactions to the orientation of the reacting atoms. *Bioorganic Chem.*, **1**, 129–139.

14. Mazor, M.H., Wong, C.F., McCammon, J.A., Deutch, J.M. and Whitesides, G. (1990) Effective molarity in diffusion-controlled reactions. *J. Phys. Chem.*, **94**, 3807–3812.

15. Zheng, X. and Vologodskii, A. (2009) Theoretical Analysis of Disruptions in DNA Minicircles. *Biophys. J.*, **96**, 1341–1349.

16. Sivak, D.A. and Geissler, P.L. (2012) Consequences of local inter-strand dehybridization for large-amplitude bending fluctuations of double-stranded DNA. *J. Chem. Phys.*, **136**, 045102.

17. Wiggins, P.A., Phillips, R. and Nelson, P.C. (2005) Exact theory of kinkable elastic polymers. *Phys. Rev. E*, **71**, 021909.
